# Complete chloroplast genome of the desert date (*Balanites aegyptiaca* (L.) Del. comparative analysis, and phylogenetic relationships among the members of Zygophyllaceae

**DOI:** 10.1186/s12864-022-08850-9

**Published:** 2022-08-31

**Authors:** Widad S. AL-Juhani, Samah A. Alharbi, Nora M. Al Aboud, Ashwaq Y. Aljohani

**Affiliations:** 1 Department of Biology, Faculty of Applied Science, Umm Al-Qura University, Makkah, 24381 Saudi Arabia; 2 Research Laboratories Centre, Faculty of Applied Science, Umm Al-Qura University, Makkah, 24381 Saudi Arabia

**Keywords:** Plastome, *Balanites aegyptiaca*, Zygophyllaceae, Phylogenetic relationship, Genome structure, Comparative analysis

## Abstract

**Background:**

*Balanites aegyptiaca* (L.) Delile, commonly known as desert date, is a thorny evergreen tree belonging to the family Zygophyllaceae and subfamily Tribuloideae that is widespread in arid and semiarid regions. This plant is an important source of food and medicines and plays an important role in conservation strategies for restoring degraded desert ecosystems.

**Results:**

In the present study, we sequenced the complete plastome of *B. aegyptiaca*. The chloroplast genome was 155,800 bp, with a typical four-region structure: a large single copy (LSC) region of 86,562 bp, a small single copy (SSC) region of 18,102 bp, and inverted repeat regions (IRa and IRb) of 25,568 bp each. The GC content was 35.5%. The chloroplast genome of *B. aegyptiaca* contains 107 genes, 75 of which coding proteins, 28 coding tRNA, and 4 coding rRNA.

We did not observe a large loss in plastid genes or a reduction in the genome size in *B. aegyptiaca,* as found previously in some species belonging to the family Zygophyllaceae. However, we noticed a divergence in the location of certain genes at the IR-LSC and IR-SSC boundaries and loss of *ndh* genes relative to other species. Furthermore, the phylogenetic tree constructed from the complete chloroplast genome data broadly supported the taxonomic classification of *B. aegyptiaca* as belonging to the Zygophyllaceae family. The plastome of *B. aegyptiaca* was found to be rich in single sequence repeats (SSRs), with a total of 240 SSRs.

**Conclusions:**

The genomic data available from this study could be useful for developing molecular markers to evaluate population structure, investigate genetic variation, and improve production programs for *B. aegyptiaca*. Furthermore, the current data will support future investigation of the evolution of the family Zygophyllaceae.

**Supplementary Information:**

The online version contains supplementary material available at 10.1186/s12864-022-08850-9.

## Introduction

The Zygophyllaceae family includes approximately 22 genera and 230–240 species [1, 2]. *Balanites aegyptiaca* (L.) Delile, desert date or heglig, belonging to the Zygophyllaceae family, is native to dry and semiarid regions in Asia and Africa. It is a thorny evergreen shrub or small tree with multiple branches, grey bark [3, 4], compound leaves, hermaphrodite flowers that are usually greenish-yellow, and brown fruit with a hardstone seed [4].

*B. aegyptiaca* has been the subject of various classic studies because of its importance as a source of food and medicines. In traditional medicine, the fruit of this plant is used to treat diabetes, asthma, epilepsy, and malaria [5]. The seeds of *B. aegyptiaca* fruit have a high concentration of oils (46.0–54.7%), especially unsaturated fatty acids (up to 75% of total fatty acids), and proteins (26.1–34.3%). The oil extracted from *B. aegyptiaca* can potentially be used as biodiesel, an alternative to chemical diesel [5]. Furthermore, *B. aegyptiaca* plays a key role in conservation strategies owing to its resistance to drought, and it has been used as a native plant in the restoration of a degraded ecosystem in Africa [4–6]. In the Great Green Wall (GGW) project, *B. aegyptiaca* was selected as one of the native plants that was convenient for restoring degraded Sahelian ecosystems. The GGW project aimed to plant a green belt of trees extending south of the Sahara Desert across 11 countries [5].

Despite the ability of plants in the Zygophyllaceae family to adapt to harsh conditions, the stability and natural diversity of *B. aegyptiaca* currently face threats from anthropogenic pressure (wood is used as a fuel), animal overgrazing, and environmental pressure due to the increasing occurrence of drought episodes [5]. Civil wars and consequent instability also represent a worrisome factor, as the migration of people negatively impacts certain areas, which become vulnerable to the depletion of natural resources [7]. In addition, *B. aegyptiaca* faces deterioration when large areas of virgin land are urbanised [8].

Historically, the genus *Balanites* has undergone numerous changes in name and taxonomic position. The species *B. aegyptiaca* was first described by Alpino in 1592 under the name Agihalid [9]. In 1753, Linnaeus described it as *Ximenia aegyptiaca*, while in 1813, Delile replaced the name Agihalid with *Balanites*, from a Greek word meaning “the fruit” [10]. Harms in1904 [11] proposed keeping the name *Balanites*, and *Balanites aegyptiaca* was formally adopted at the Vienna Botanical Congress in 1905 [9]. Initial classifications in the genus *Balanites* were based on morphological characteristics and were vulnerable to conflict, which led to the movement of *Balanites* between plant families. Initially, Bentham in 1862 [12] placed it within the family Simaroubaceae, while Engler 1896 [13] moved it to the family Zygophyllaceae. Cronquist in 1968 [14] returned it again to Simaroubaceae. Hegnauer in 1973 [15] provided evidence that *Balanites* did not contain quassia-like alkaloids, which are the main chemical characteristic of the family Simaroubaceae. Hegnauer 1973, Scholz 1964 and Cronquist 1981 [15–17] supported the return of *Balanites* to the Zygophyllaceae family again. Maksoud 1988 [18] mentioned that similarities between the flavonoids of *Balanites* and Zygophyllaceae did not support the treatment of *Balanites* as a separate family. Sheahan 1993 [19] studied 37 species in 19 genera within Zygophyllaceae. Based on anatomy and c4 activity in 27 species, the results of their study supported the separation of *Balanites* into an independent family named Balanitaceae. Boesewinkel 1994 [20] also supported the separation of *Balanites* in a special family based on distinguishing features of ovule and seed characters. Next, the molecular and anatomical characteristics of flowers and embryos, in addition to pollen characteristics, supported the classification of *Balanites* within the family Zygophyllaceae [21, 22] . However, he last comprehensive review performed by [23] supported the separation of *Balanites* and its placement in the family Balanitaceae.

While a few molecular phylogenetic studies have been conducted for the Zygophyllaceae family, no study has specifically addressed *B. aegyptiaca*. The molecular aspects of Zygophyllaceae were studied by [24] using the plastid gene *rbcL*, in combination with anatomical and morphological data from 20 Zygophyllaceae species, including *B. maughamii*. Based on morphological and phylogenetic data, the redistribution of Zygophyllaceae into five subfamilies, namely, Morkillioideae, Tribuloideae, Seetzenioideae, Larreoideae, and Zygophylloideae, was proposed [24]. Recently, [1] presented a phylogenetic tree for Zygophyllaceae based on the Bayesian analysis tree of combined sequence data *(rbcL, trnL-F,* and *ITS*) for these five subfamilies. The results were consistent with previous studies [21, 22, 24, 25], and the *Balanites* genus was affiliated with the Tribuloideae subfamily within the Zygophyllaceae family. On the other hand, [26] reported that *B. aegyptiaca* includes five varieties; however, this number was later reduced to two by [26]. In a study by [27], three ecotypes were reported within the *B. aegyptiaca* population in Egypt based on RAPD markers. This discrepancy in the number of varieties belonging to *Balanites aegyptiaca* may be due to variation in morphological characteristics affected by environmental conditions.

Chloroplasts, which have an essential role in photosynthesis, are organelles in plant cells that contain their own genome, the plastome [28]. Typically, chloroplast genome sizes range between 120 and 170 kilobase pairs (kb) [29]. A chloroplast genome presents a four-region structure comprising a large single copy (LSC), a small single copy (SSC), and two inverted repeats (IRa and IRb). During the evolutionary history of plant families, plastomes have been subjected to strong selective pressure [30]. Thus, chloroplast genomes include useful phylogenetic information that can be used to study evolutionary relationships at different taxonomic levels and resolve difficult problems in plant phylogenetics. In addition, chloroplast genomes represent a database for identifying and developing efficient polymorphic molecular markers for studying genetic diversity and population structure, and for DNA barcoding, which is a tool for identification [31, 32].

Zygophyllaceae is an angiosperm family that utilizes the C4 pathway [33], which helps plants adapt to harsh, dry environments. Molecular phylogenetic analyses suggest that the evolutionary history of Zygophyllaceae is related to an arid period that began in the Oligocene in Asia and Africa [34, 35]. The most recent study of three chloroplast genomes of *Zygophyllum* species belonging to the Zygophyllaceae family reported a significant reduction in the genome size of these species [36]. In this study, we validated the hypothesis that dry environments will significantly reduce the genome size of *B. aegyptiaca* in the family Zygophyllaceae. The aims of the present study are to provide baseline molecular information on the *B. aegyptiaca* plastome (*B. aegyptiaca* is considered typical of plants in dry and semiarid environments), to verify the plastome structure of *B. aegyptiaca*, and to perform comparative analyses and investigate phylogenetic relationships and variations between *B. aegyptiaca* and related Zygopyllaceae species, based on an available complete plastome dataset in GenBank.

## Results

### Characteristics of the *B. aegyptiaca* chloroplast Genome

**Fig. 1:**
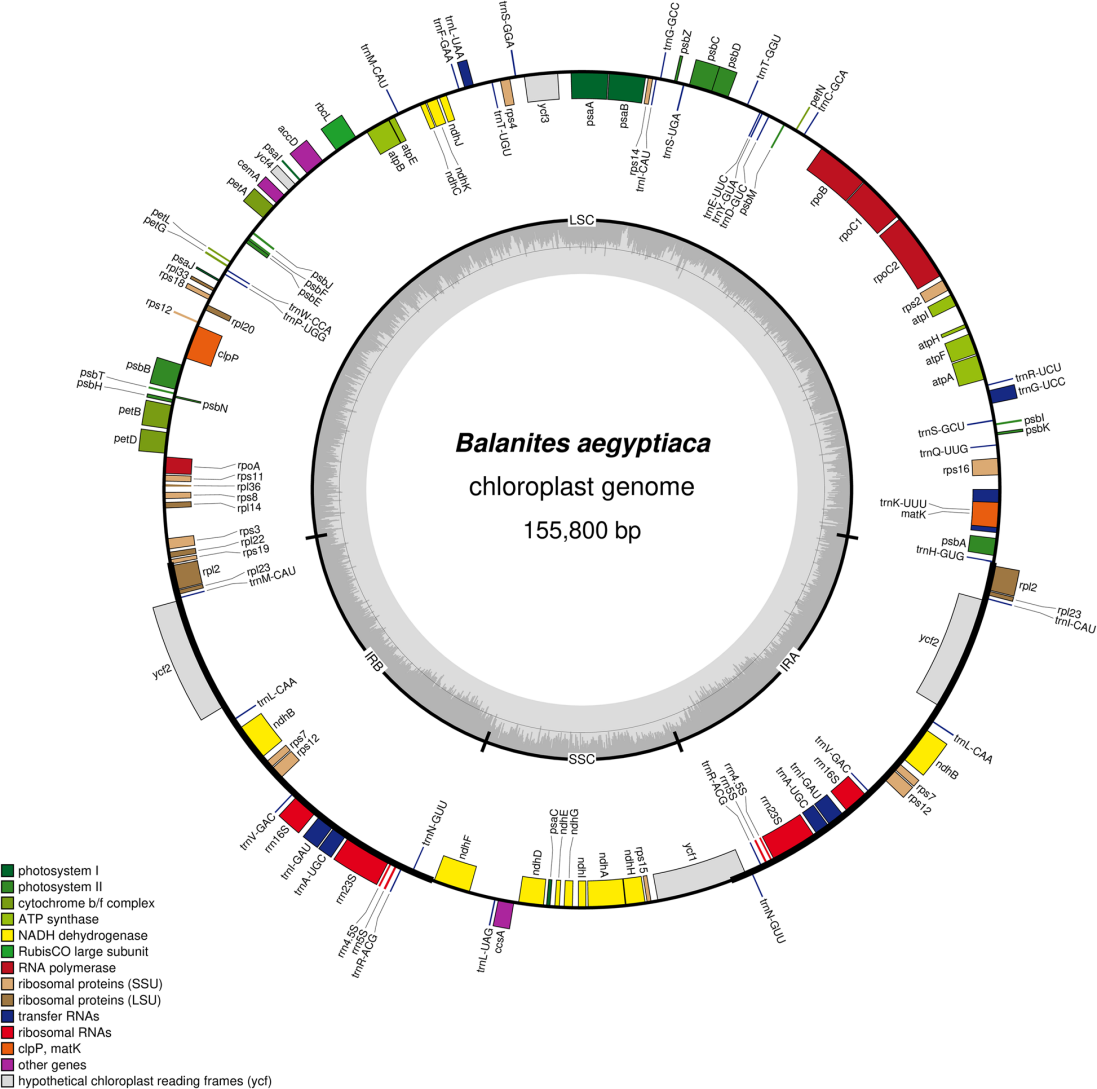
Gene map of *Balanites aegyptiaca* chloroplast genome. Genes outside the circles are transcribed counter clockwise, while those inside are transcribed clockwise. Known functional genes are indicated in the coloured bar

The cp genome of *B. aegyptiaca*, shown in Fig. 1, is a circular molecule with a length of 155,800 bp. It has a four-region structure comprising a large single copy, a small single copy, and two inverted repeats. The LSC and SSC regions were 86,562 bp and 18,102 bp long, respectively, while IRa and IRb regions were 25,568 bp each (Table 1). The length of the coding region is 75,890 bp and represents 49% of the whole genome, while the noncoding region length is 79,910 bp (51%). The percentage of AT in the whole genome was 64.5%, whereas the percentage of GC was 35.5%. The genome of *B. aegyptiaca* consists of A = 31.8%, T(U) = 32.7%, C = 18.1% and G = 17.4%, as shown in Table 1.

**Table 1:** Chloroplast genome features of *Balanites aegyptiaca*

Feature	***B. aegyptiaca***
Genome size (bp)	155,800
IRA (bp)	25,568
IRB (bp)	25,568
LSC (bp)	86,562
SSC (bp)	18,102
Total No of Unique Genes	107
rRNA	4
tRNA	28
Protein-Coding genes	75
A%	31.8
T (U)%	32.7
G%	17.4
C%	18.1
GC%	35.5

The total number of unique genes was 107, and the number of duplicated genes in the inverted region was 18. The LSC region was composed of 60 protein-coding genes and 21 tRNA genes, the SSC region contains 11 protein-coding genes and one tRNA, and 10 protein-coding genes and 22 tRNAs are located in the IR regions. Most of the protein-coding genes start with a methionine codon (AUG).

Sixteen of the 107 genes in *B. aegyptiaca* contain introns, 11 are protein coding genes, and five were tRNA genes, as illustrated in Table 2. The *clpP* and *ycf3* genes present two introns, while the remaining genes present only one intron. Ten introns were included in the LSC region, one intron is included in the SSC region, and five introns were specifically located within the IRa and IRb regions.

**Table 2:** Genes with introns in the chloroplast genome of *Balanites aegyptiaca*

Gene	Location	Exon I (bp)	Intron I (bp)	Exon II (bp)	Intron II (bp)	Exon III (bp)
*rpoC1*	LSC	432	798	1617		
*rpl2*	IRA	394	665	434		
		434	665	394		
*ndhA*	SSC	539	1165	553		
*ycf3*	LSC	153	793	230	733	124
*clpP*	LSC	247	634	294	836	71
*petB*	LSC	6	785	642		
*petD*	LSC	8	780	477		
*rps16*	LSC	228	757	40		
*atpF*	IRA	410	736	145		
*ndhB*	IRB	758	679	775		
		775	679	758		
*rps12*	LSC	114		26	538	232
		114		232	538	26
*trnL-UAA*	LSC	35	493	50		
*trnK-UUU*	LSC	35	2537	37		
*trnI-GAU*	IRB	32	956	40		
		40	956	32		
*trnA-UGC*	IRB	37	802	36		
		36	802	37		
*trnG-UCC*	LSC	32	715	60		

### Relative synonymous codon usage (RSCU)

**Fig. 2:**
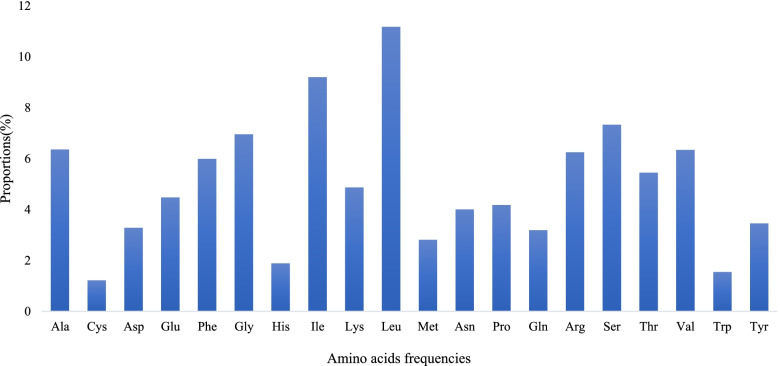
Amino acid frequencies of the protein-coding sequences of *Balanites aegyptiaca* chloroplast genome

The nucleotides of protein coding and tRNA genes were used to determine the codon usage bias of the plastome. The results obtained from the analysis of protein-coding genes and tRNA genes (78,624 bp) of the *B. aegyptiaca* plastome are shown in Table S2. Genes are encoded by 22,415 codons. Leucine is the most frequent amino acid (11.1%), as shown in Fig. 2, whereas cysteine is the least frequent (1.2%). The RSCU values in Table S2 show that half (30) of the codons are > 1, all with an A/T ending. It can be seen from the data that tryptophan and methionine with no codon usage bias have an RSCU value of 1.

### RNA editing sites

The PREP suite was used to predict the RNA editing sites in the *B. aegyptiaca* plastome, and the first codon position of the first nucleotide was used in the analysis.

The RNA editing sites are presented in Table S3. Overall, there were 41 editing sites in the genome distributed among protein-coding genes. Most of the codon position exchanges involved serine (S) and leucine (L) amino acids (S to L). The results obtained show the highest number of editing sites in the *ndhB* and *ndhD* genes (12 and 6 sites, respectively), followed by *rpoB* and *ndhF* genes with three sites. Moreover, the *accD, atpA, matK, and ndhA* genes have 2 sites, while 1 site is present for each of the remaining genes. The results of RNA editing show that certain genes, namely, *atpI, ccsA, petB, petD, petG, petL, psaB, psaI, psbB, psbE, psbF, rpl2, rpl23, rpoA, ycf3,* and *atpB*, do not possess a predicted site in the first codon of the first nucleotide*.*

### Repeat Analysis

#### Long repeats

There are four types of repeats in the cp genome of *B. aegyptiaca*: palindromic (21), forward (12), reverse (15), and complement (1), as evident from Table S4. Overall, there were 49 repeats in the *B. aegyptiaca* plastome. The majority (75.5%) of repeats were found in the intergenic spacer (IGS) region. The sizes of most of the repeats range from 20 to 29 bp (71.4%), followed by 10–19 bp (14.3%), 30–39 bp (10.2%), and 40–49 bp (4.1%). The tRNAs include 4 repeats (8.2%), while the remaining 8 repeats (16.3%) were in the protein-coding genes *ndhC, ycf2, clpP, ycf1,* and *ndhA*. We noticed that the protein-coding gene *ycf2* had the most repeat locations: 2 palindromic repeats and 2 forward repeats.

**Fig. 3:**
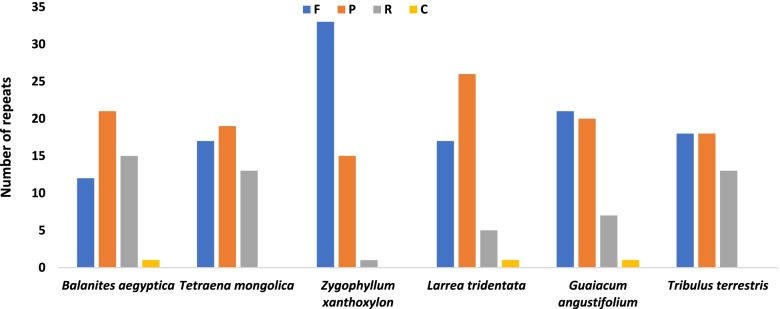
Number of different repeats among six Zygophyllaceae plastomes. F, forward repeats; P, palindromic repeats; R, reverse repeats; and C, complement repeats

A comparison of the number of repeats in six Zygophyllaceae species (*Balanites aegyptiaca* (L.), *Guaiacum angustifolium* Engelm*., Larrea tridentata* (DC.) Coville*, Tetraena mongolica* Maxim.*, Tribulus terrestris* L.*, and Zygophyllum xanthoxylon* (Bunge) Maxim.) is provided in Fig. 3 (species description is available in Table S5); the genome of *L. tridentata* has the highest frequency of palindromic repeats (26), while that of *Z. xanthoxylon* has the lowest (15). *L. tridentata* and *T. mongolica* have the same number of forward repeats (17), while the same number of reverse repeats occur in the cp genome of *T. mongolica* and *T. terrestris* (13). We can also see that complement repeats are the least abundant in the three genomes; *B. aegyptiaca, L. tridentata* and *G. angustifolium* have only one complement repeat, and complement repeats are absent in the other three species.

#### Simple sequence repeats (SSRs)

**Fig. 4:**
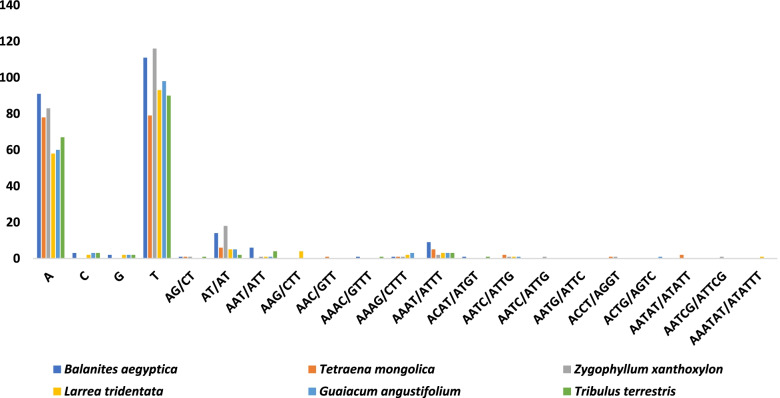
Frequency of different microsatellite motifs in different repeat types of six Zygophyllaceae plastome genomes

**Fig. 5:**
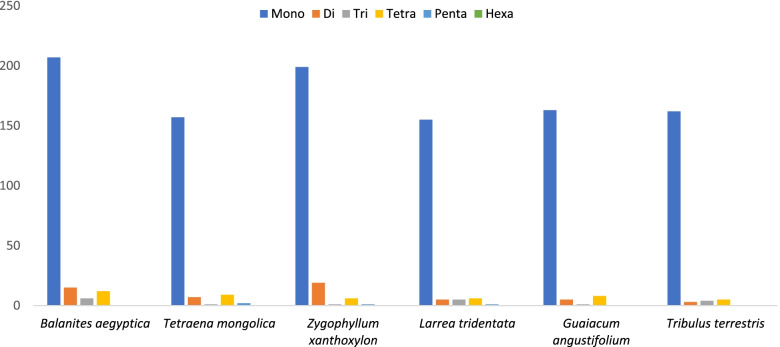
Number of different SSR types in the six Zygophyllaceae chloroplast genomes

A total of 240 SSRs are present in the plastid genome of *B. aegyptiaca*, which is a larger number than in the five other species of Zygophyllaceae shown in Table 3. The majority of SSRs in the cp genome are mononucleotides (86. 3%), mostly poly T (53.6%) and A (44%) (Figs. 4 and 5); poly C and G represent 1.5 and 1% of repeats, respectively. The results obtained from the analysis of SSRs frequency in the genomes of six species of Zygophyllaceae are presented in Table 3 and Fig. 4. The dinucleotide AT/AT was found in the genome of all species, while the dinucleotide AG/CT was found in four species but was absent from *L. tridentata* and *G. angustifolium*. Furthermore, the existence of three trinucleotides (AAT/ATT, AAG/CTT, and AAC/GTT), as well as nine tetra-repeats (AAAC/GTTT, AAAG/CTTT, AAAT/ATTT, ACAT/ATGT, AATC/ATTG, AATC/ATTG, AATG/ATTC, ACCT/AGGT, and ACTG/AGTC), and three pentanucleotides (AATAT/ATATT, AATCG/ATTCG, and AAATAT/ATATTT) was observed only in *T. mongolica, Z. xanthoxylon,* and *L. tridentata* (Fig. 4).

**Table 3:** cpSSRs detected in six Zygophyllaceae chloroplast genomes

SSR type	Repeat unit	*Balanites aegyptiaca*	*Tetraena mongolica*	*Zygophyllum xanthoxylon*	*Larrea tridentata*	*Guaiacum angustifolium*	*Tribulus terrestris*
Mono	A	91	78	83	58	60	67
	C	3	0	0	2	3	3
	G	2	0	0	2	2	2
	T	111	79	116	93	98	90
Di	AG/CT	1	1	1	0	0	1
	AT/AT	14	6	18	5	5	2
Tri	AAT/ATT	6	0	1	1	1	4
	AAG/CTT	0	0	0	4	0	0
	AAC/GTT	0	1	0	0	0	0
Tetra	AAAC/GTTT	1	0	0	0	0	1
	AAAG/CTTT	1	1	1	2	3	0
	AAAT/ATTT	9	5	2	3	3	3
	ACAT/ATGT	1	0	0	0	0	1
	AATC/ATTG	0	2	1	1	1	0
	AATC/ATTG	0	0	1	0	0	0
	AATG/ATTC	0	0	0	0	0	0
	ACCT/AGGT	0	1	1	0	0	0
	ACTG/AGTC	0	0	0	0	1	0
Penta	AATAT/ATATT	0	2	0	0	0	0
	AATCG/ATTCG	0	0	1	0	0	0
	AAATAT/ATATTT	0	0	0	1	0	0
Total		240	176	226	172	177	174

A comparison between the frequency of SSRs in the plastomes of the six species is presented in Fig. 5; it is clear that mononucleotides are the most frequent in all genomes. *B. aegyptiaca* had the highest number of mononucleotides, trinucleotides, and tetranucleotides (207, 6, and 12, respectively). However, pentanucleotides were not present in the cp genomes of *B. aegyptiaca, G. angustifolium,* or *T. terrestris.* In addition, hexa-repeats were not present in any of the six species.

**Fig. 6:**
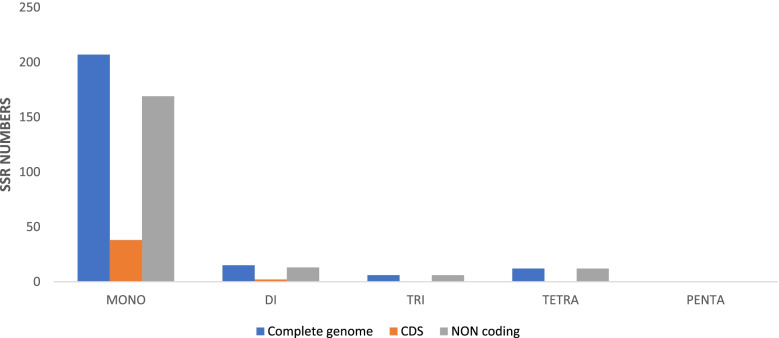
Number of SSR types in the complete chloroplast genome, protein-coding regions, and non-coding genes of *Balanites aegyptiaca*

Overall, the IGS region included most of the SSRs repeats (83.3%), shown in Fig. 6, followed by the coding regions (16.7%).

### Sequence divergence

**Fig. 7:**
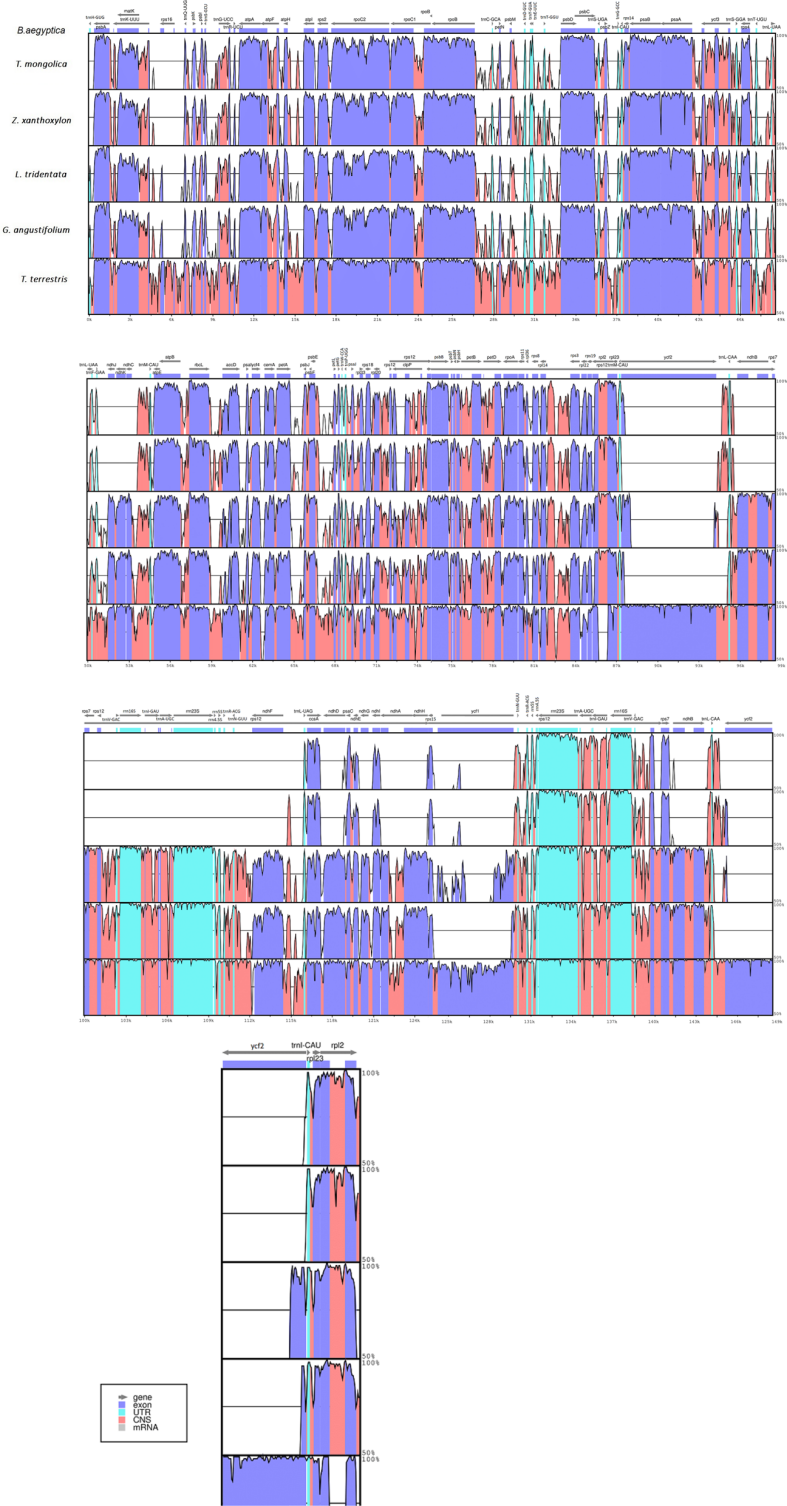
Whole chloroplast genome alignments for six Zygophyllaceae species via the mVISTA program, using the annotation of *Balanites aegyptiaca* as reference. The x-axis represents the coordinates in the cp genome, while the y-axis indicates percentage identity from 50 to 100%. The top grey arrows indicate the position and direction of each gene. Pink indicates non-coding sequences (NCS), blue indicates protein-coding genes, and light green indicates tRNAs and rRNAs

To investigate the degree of genome sequence divergence, the program mVISTA was used to align the cp genome sequence of *B. aegyptiaca* with five Zygophyllaceae chloroplast genomes available in GenBank: *T. mongolica, Z. xanthoxylon, L. tridentata, G. angustifolium,* and *T. terrestris*. The alignment showed that genes were less conserved in the genomes of *T. mongolica* and *Z. xanthoxylon*, especially the *ndh* genes *rps16* and *ycf2*. In general, the protein-coding genes were more conserved than the noncoding regions (Fig. 7). The noncoding regions presented high divergence in the following genes: *psabA-trnK-UUU, trnK-UUU-rps16, psbK-psbI, trnG-UCC-trnR-UCU, atpF-atpH, atpH-atpI, rps2-rpoC2, rpoC2-rpoC1, rpoC1-rpoB, rpoB-trnC-GCA, psbD-trnS-UGA, psaA-ycf3, ycf3-trnS-GGA, trnS-GGA-rps4, trnT-UGU-trnL-UAA, ndhC-trnM-CAU, ntrnM-CAU-atpE, atpB-rbcL, rbcL-accD, cemA-petA, petA-psbJ, psbJ-psbF, psbE-petL, psaJ-rpl33, rps18-rpl20, rps12-psbB, psbB-psbT, psbH-petB, petB-petD, petD-rpoA, rps8-rpl14, rpl14-rps3, rpl2-rpl23, ycf2-trnL-CAA, trnL-CAA-ndhB, ndhF-trnL-UAG, psaC-ndhE, trnN-GUU-trnR-ACG, trnA-UGC-trnI-GAU, trnI-GAU-rrn16S, rrn16S-trnV-GAC, trnL-CAA-ycf2, trnI-CAU-rpl23,* and *rpl23-rpI2*. However, the following protein-coding genes showed divergence in fewer regions: *matK, rpoC2, psaA, accD, cemA, clpP, rpl23, ndhF, ccsA,* and *rpl23*.

### Boundary between LSC/SSC and IR regions

**Fig. 8:**
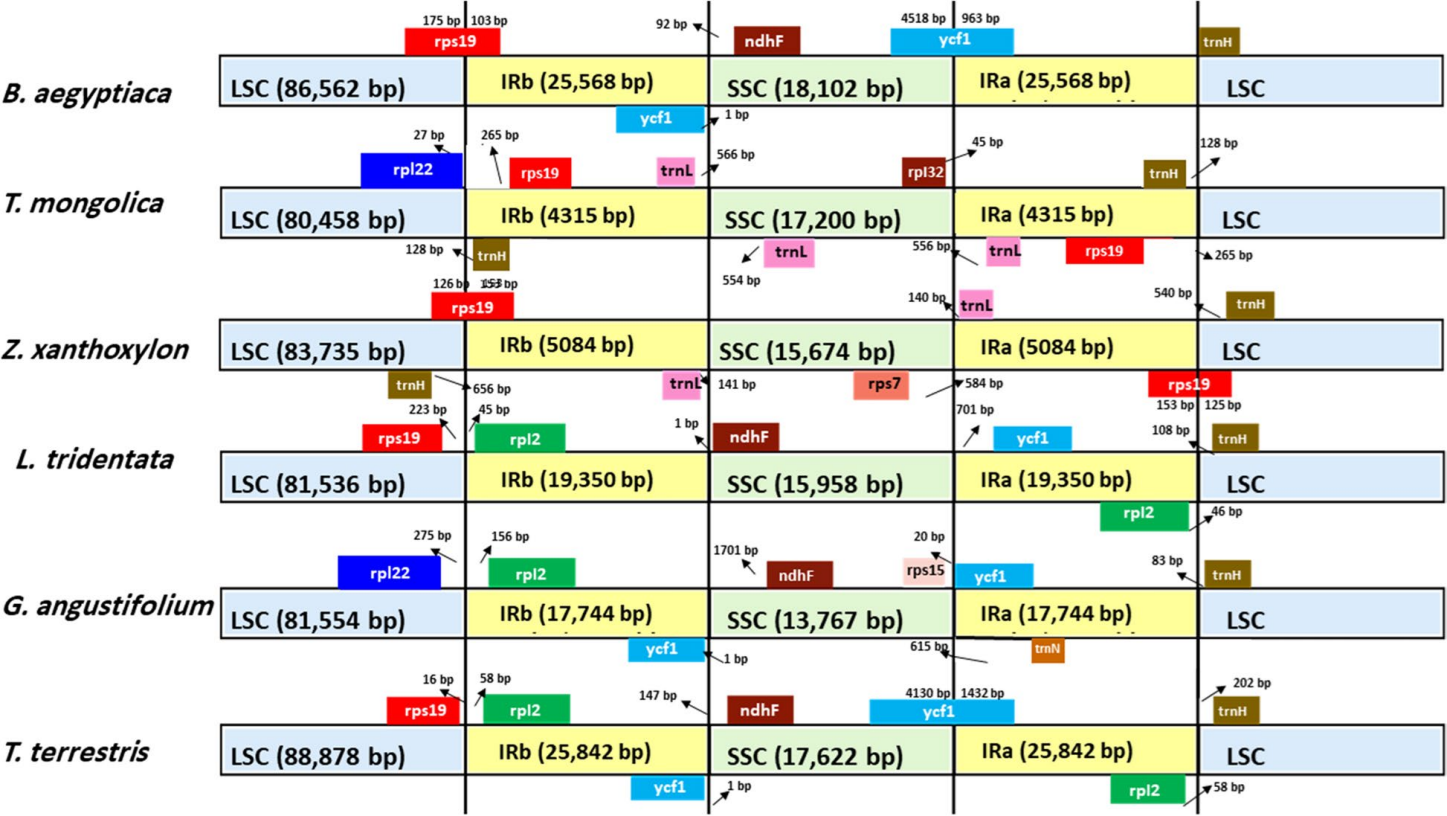
Comparison of the LSC, SSC, and IR region borders among the chloroplast genomes of six Zygophyllaceae species

The comparisons between IR-SC boundaries for the six species of Zygophyllaceae are shown in Fig. 8. In general, the variation in length of the two LSC/SSC regions was lower than that of the IRa/IRb regions. The shortest LSC and SSC regions were 80,458 bp and 13,767 bp, respectively, in *T. mongolica* and *G. angustifolium*, while the longest LSC and SSC regions were 88,878 bp and 18,102 bp, in *T. terrestris* and *B. aegyptiaca*, respectively. The IRa/IRb regions of *B. aegyptiaca* and *T. terrestris* were the longest (25,568/25842), and contraction was noticed in the IR region in both *Z. monogolica* (4315 bp) and *Z. xanthoxylon* (5084 bp) compared to the other species (Table S6).

The *ycf1* gene was located within the SSC/IR border in *B. aegyptiaca, T. terrestris,* and *G. angustifolium* but was located within the IRa region in *L. tridentata* (701 bp). The pseudogene *ycf1* was present in the IRb/SSC border in *B. aegyptiaca, G. angustifolium* and *T. terrestris.* In contrast, the *ycf1* gene was not present in *Z. xanthoxylon* or *T. mongolica*; instead, the *rpl32* gene was located in the SSC region, close to the SSC/IRa border (45 bp) in *T. mongolica*, and the *rps7* gene was present in the SSC region in *Z. xanthoxylon*. Both *T. mongolica* and *Z. xanthoxylon* possessed two copies of the *trnL* gene (in the IRa and IRb regions).

The *ndhF* gene was in the IRb/SSC border of *L. tridentata* (1 bp), whereas gene *ndhF* was in the SSC (92 bp) of IRb/SSC border in *B. aegyptiaca* and *T. terrestris* (147 bp), while *G. angustifolium* having *ndhF* gene in (1701 bp) of the IRb/SSC border. However, the *ndhF* gene was missing from the SSC region in both *Z. xanthoxylon* and *T. mongolica* as well.

The *trnH* gene showed variation in its location in the IR/LSC border. *B. aegyptiaca* had the *trnH* gene in the IRa/LSC border, while it was located in the LSC region and far from the IRb/LSC border in *Z. xanthoxylon* (540 bp), *L. tridentata* (108 bp), *G. angustifolium* (83 bp), and *T. terrestris* (202 bp). *T. mongolica* was different; the *trnH* gene was located in the IRa region far from the IRa/LSC border (128 bp). *T. mongolica* and *Z. xanthoxylon* had another copy of the *trnH* gene; it was present in the IRb region and far from the IRb/SSC border (128 bp) in *T. mongolica*, and it was in the LSC region far from the IRb/SSC border (656 bp) in *Z. xanthoxylon.*

Variations in the location of the *rps19* gene in the IR/LSC border also occurred, with it being located at different sites in the cp genomes (Fig. 8). The *rps19* gene spanned the border of LSC/IR in *B. aegyptiaca* and *Z. xanthoxylon. Z. xanthoxylon* duplicated the *rps19* gene in the IRa region. *rps19* was located in the LSC region, distant to the LSC/IR border in *L. tridentata* (223 bp) and *T. terrestris* (16 bp). Both species possessed duplicated *rpl2* genes in the IRa and IRb regions. *T. mongolica* had two copies of the *rps19* gene located in the inverted repeat region (IRa and IRb), whereas the *rpl22* gene was in the LSC region. The *rpl22* and *rpl2* genes were located in the LSC and IRa regions in *G. angustifolium*, respectively.

### Characterisation of substitution rate

**Fig. 9:**
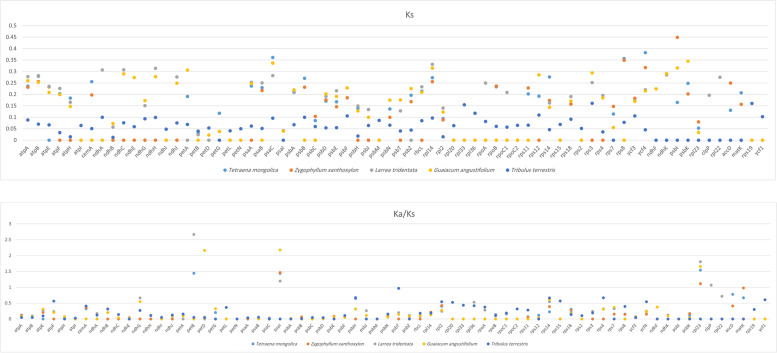
The synonymous (Ks) and Ka/Ks ratio values of 70 protein-coding genes of the six Zygophyllaceae plastomes

The value of synonymous (Ks) and nonsynonymous (Ka) substitutions and the Ka/Ks ratio were calculated among the 70 protein-coding genes that represent the common genes in the species that were compared within Zygophyllaceae (*T. mongolica, Z. xanthoxylon, L. tridentata, G. angustifolium,* and *T. terrestris*). Several genes were under positive selection with Ka/Ks values > 1, as shown in Fig. 9: *atpF, ndhG, petB, petD, psaI, psbH, psbT, rpl2, rps14, rps4, rps7, ycf4, rpl23,* and *matK*. In addition, most Ks values were < 1 in all genes (Fig. 9), except for *atpA, atpB, psaC, rpl14,* and *ycf*4.

### Phylogenetic Analysis

**Fig. 10:**
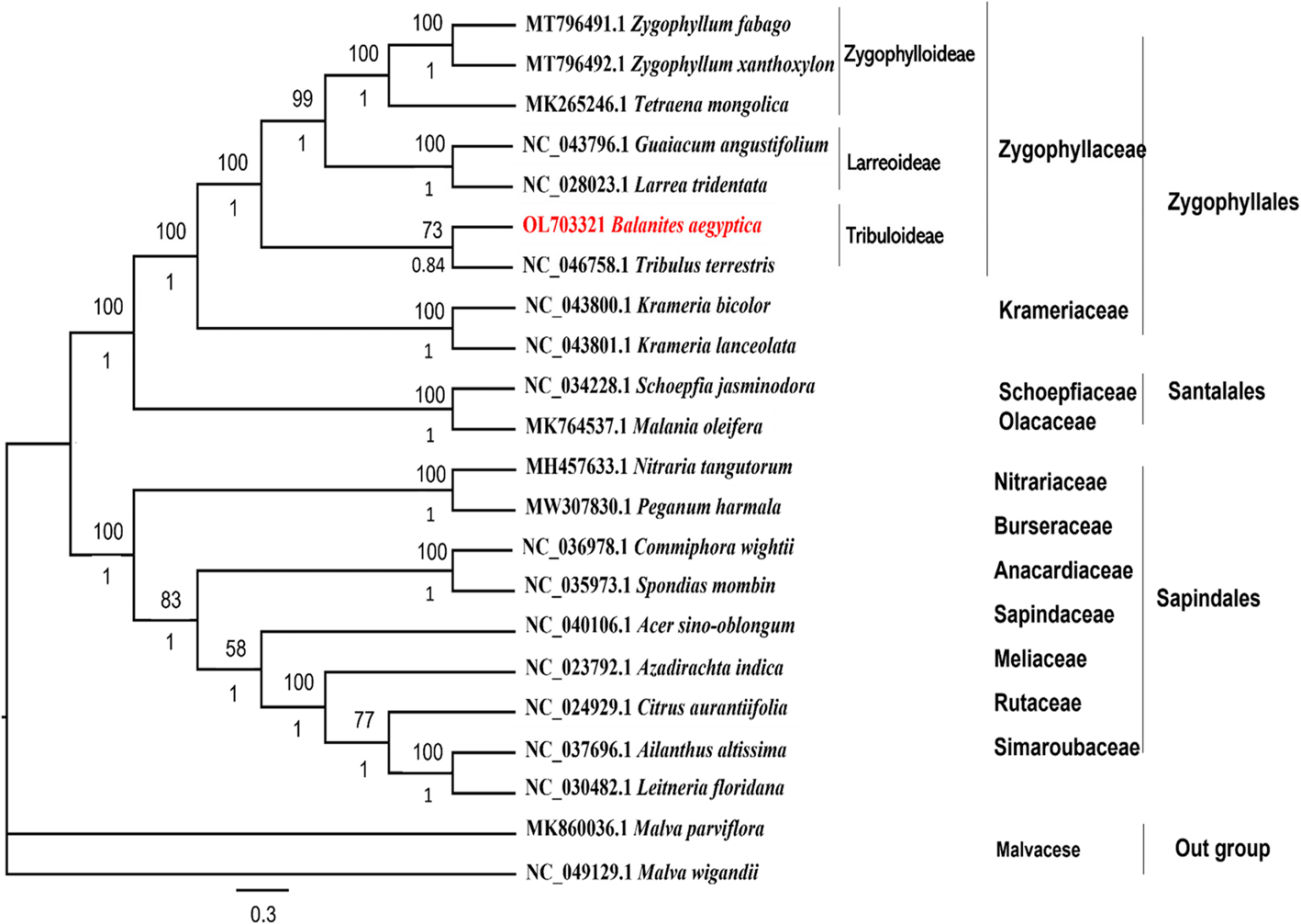
Phylogenetic tree construction inferred from the coding sequence (CDS) of 20 taxa, using Bayesian Inference (BI) and Maximum Parsimony (MP) methods. The tree shows the relationships between Zygophyllales (Krameriaceae & Zygophyllaceae), Sapindales, and Santalales. The numbers in the branch nodes represent bootstrap (BP) values of Bayesian posterior probabilities (PP)

Phylogenetic relationships were assessed among all currently available cp genome sequences in GenBank for the order Zygophyllales (six species from the family Zygophyllaceae), two species from Krameriaceae, and 11 plastome sequences from the orders Sapindales and Santalales, where *B. aegyptiaca* was classified formerly. Phylogenetic analysis was performed via MP and BI analyses. The two phylogenetic trees were topologically similar, with the majority of nodes having 100% bootstrap (BP) values and 1.00 Bayesian posterior probabilities (PP) (Fig. 10). The monophyly of the Zygophyllales order is strongly supported (PP = 1.00; BS = 100%) by this study.

Zygophyllales was divided into two highly supported clades (PP = 1.00; BS = 100%): one clade contained species from the Krameriaceae family, and the other comprised Zygophyllaceae species. Zygophyllaceae was further divided into three separate clades representing three subfamilies: Tribuloideae (PP = 0.84; BS = 73%), Larreoideae PP = 1.00; BS = 100%), and Zygophylloideae (PP = 1.00; BS = 100%). In this study, *B. aegyptiaca* was nested in the Zygophyllaceae family and formed a moderately supported clade with *T. terrestris*, representing the Tribuloideae subfamily.

## Discussion

This study presents the first chloroplast genome from *B. aegyptiaca*, a plant within the Tribuloideae (Zygophyllaceae) subfamily. The length of the cp genome in *B. aegyptiaca* (155 kb) was similar to that seen in the cp genome of *Tribulus terrestris* (158 kb), which is also within the Tribuloideae subfamily. Previous studies have [36] indicated a great reduction in plastid genome size in three species of the subfamily Zygophylloideae (Zygophyllaceae), namely, *T. mongolica, Z. xanthoxylon,* and *Z. fabago*. The sizes of these genomes ranged between 104 and 106 kb. In the current study, a large decrease in the size of the *B. aegyptiaca* genome, which has mentioned in regard to some Zygophyllaceae species, was not observed [36]. Size, structure, gene content, and organization are usually conserved in the chloroplast genomes of angiosperms [30]. However, the chloroplast genomes in some species are smaller than those in most other angiosperms. The cp genome in Saguaro (*Carnegiea giganteais*) considered to the smallest known plastid genome in autotrophic angiosperms having size (113 kb), where lost whole of IR region and *ndh* genes [37]. As well in *Astragalus membranaceus* of the Fabaceae whose chloroplast genome size is 124 kb [38]. In addition, to decrease in genome size is a common feature of parasitic plants and is caused by their conversion from an autotrophic to parasitic strategy. This decrease in genome size is accompanied by several other changes, such as pseudogenization, gene loss, structural rearrangement, and size reduction [39, 40]. For example, the plastid genome size in parasitic species of the Loranthaceae family ranges from 116 to 139 kb and the reductions in the plastid sizes of some holoparasites may be even greater. Indeed, the root parasite species in the family Cynomoriaceae has a total plastid genome length of 45,519 bp [41].

The organization, size, and structure of the *B. aegyptiaca* chloroplast genome is similar to those of other angiosperms; the size of the LSC region is 86 kb, the SSC region is 18 kb, and the two IR regions are 25 kb. The LSC region in angiosperms ranges from 80 to 90 kb, SSC regions are approximately 16–27 kb, and the sizes of the two IRs range from 20 to 28 kb [36].

A typical angiosperm chloroplast genome consists of 113 genes, including 79 protein coding genes, 30 tRNA genes and four rRNA genes [30]. The *B. aegyptiaca* chloroplast genome had a similar number of genes (107 genes), including 75 protein-coding genes, 28 tRNA genes and 4 rRNA genes. This was similar to other species in the *Zygophyllum* genus of the family Zygophyllaceae [36], which contained 107 genes, including 75 protein-coding genes, 33 tRNA genes and 4 rRNA genes.

We found that a GC content of 35% in the *B. aegyptiaca* cp genome was akin to GC content in *Zygophyllum* spp. [36], which ranged between 33 and 36%. Codon usage bias plays an important role in chloroplast genome evolution and occurs as a result of natural selection and mutations [42, 43]. Codons encoding the amino acid leucine were the most frequently observed in the plastome of *B. aegyptiaca*, whereas those encoding cysteine were the least frequently observed. This finding has also been reported in the chloroplast genomes of *Populus* species [44]. While isoleucine is the most encoded amino acid among *Zygophyllum* spp. (Zygophyllaceae), methionine is a less abundant amino acid [36].

Introns play a significant role in gene expression and regulation [45, 46]. The *clpP* and *ycf3* genes in the *B. aegyptiaca* plastome had two introns, while the remaining genes contained only one intron; these findings are consistent with previously published reports [47, 48]. The chloroplast genomes of three species of *Zygophyllum* also contained two introns in the *ycf3* gene [36].

RNA editing includes the processes of inserting, deleting, or modifying nucleotides, which leads to changes in the DNA coding sequence during RNA transcription [49]; in turn, this allows the creation of different protein transcripts [50]. Only C-U RNA editing has been described in plastomes [51]. The results obtained from the present study show that in the *B. aegyptiaca* plastome, most of the transformation in the codons was from the amino acid exchange of serine to leucine and that the *ndhB* gene had the highest number of editing sites, which could be attributed to high preservation of RNA editing [52, 53].

Prior studies have noted the importance of repeat sequences in the cp genome, which play a significant role in genomic recombination [54, 55]. In the *B. aegyptiaca* plastome, most repeats are in the *ycf2* gene, which is consistent with data obtained from other angiosperms [56, 57].

Several reports [58–61] have shown the importance of chloroplast SSRs (cpSSRs) as reliable molecular markers to discriminate between specimens at lower taxonomic levels and in studying population structure. In the *B. aegyptiaca* cp genome, there are 240 SSRs repeats, the largest number compared to five other species of Zygophyllaceae. Mononucleotide A/T SSRs were the most frequent, and most SSRs were marked in noncoding regions (83.3%). Thus, we recommended used plastid genome data in this study for developing cpSSR loci and for studying the levels of genetic variation in the *B. aegyptiaca* population.

Although the plastid genome is conserved in angiosperm plants as previously reported [30], several studies have reported variations in size and boundaries among IR/LSC and IR/SSC regions and variation in gene location [62, 63]. In the present study, comparisons between the IR-LSC and IR-SSC boundaries in the six complete Zygophyllaceae chloroplast genomes showed clear variation in the inverted repeat region in Zygophyllaceae chloroplast genomes and significant contraction in the IR region in the *Z. xanthoxylon and T. mongolica* genomes (synonym of *Zygophyllum mongolicum*). Our results are consistent with recent results published by [36], who mention that the most conspicuous change occurs in two IRs reduced by approximately 16–24 kb in size in the plastid genomes of *T. mongolica, Z. xanthoxylon,* and *Z. fabago*.

There is variation in the border of the IR-SC region among the six species of Zygophyllaceae (in this study based on the position of the *rps19, rpl2* and *trnH* genes) at the LSC/IRa junction, which could occur as a result of contraction and expansion in the inverted repeat region. A similar observation of variation in the location of the *trnH, rpl2,* and *rps19* genes at the border was reported for species in the subfamily Acanthoideae by [64]. Furthermore, two copies of the *trnL* gene appeared in the IRs of both *T. mongolica* and *Z. xanthoxylon.* This could be related to IR border changes due to either gene loss, such as *ndhF*, or expansion/reduction. The *trnL* genes at the IR borders of *Tetraena mongolica* and *Zygophyllum xanthoxylon* are not the same, *trnL-CAA* and *trnL-UAG* respectively. The *trnL- CAA* is usually located in the IR regions and *trnL-UAG* in the SSC region. We noticed that the *ycf1* gene was located in the IRb/SSC border in *B. aegyptiaca, G. angustifolium* and *T. terrestris*, and studies [65] have indicated that the *ycf1* gene in the IRb region is a pseudogene in the angiosperm chloroplast genome.

In the current comparison, we observed that all the *ndh* genes that were usually located in the SSC and IR regions were lost in *T. mongolica* and *Z. xanthoxylon*. This was similar to the mVISTA results, which also showed a loss of *ndh* genes (specifically in the sequences from *T. mongolica* and *Z. xanthoxylon*). A similar loss of the *ndh* group (11 genes) from the chloroplast genomes of *T. mongolica*, *Z. xanthoxylon,* and *Z. fabago* was also reported by [36], and it was reported that rRNA genes, which are usually present in IRs, were in the SSC region. In our study, although we used plastome sequences from GenBabk with accession numbers that were different than those used by [36] for the species *T. mongolica* and *Z. xanthoxylon*, we observed a similar loss of all *ndh* genes and the presence of ribosomal proteins codes (*rpl32* & *rps7*) genes in the SSC region in Zygophyllum spp., which could be a result of contraction of the IR regionas. The *ndh* genes represent a complex group consisting of approximately 30 subunits, and 11 out of 30 subunits are used in encoding the NADH dehydrogenase complex in plant plastids and are involved in photosynthesis [66]. The partial or complete loss of genes associated with photosynthesis (*ndh*) has been reported in some species of the Cactacaeae [37], Pinaceae [67], and Orchidaceae families [68]. It is also a common phenomenon in hemiparasites and holoparasites of the Santalales and Orobanchaceae [69–74].

On the other hand, in a study by [36] on three species of the subfamily Zygophylloideae, the authors indicated a significant loss in chloroplast genes (11 genes), and it was not certain if this loss was because of adaptation by plants to living in arid and semiarid environments or as a result of gene transmission to the nuclear genome. In our study, we present the cp genome of *B. aegyptiaca* (subfamily Tribuloideae and family Zygophyllaceae), which is adapted to arid and semiarid lands. However, we did not observe a large loss in plastid genes and all 11 *ndh* genes were present in the *Balanites* plastome. Another possible explanation could be that gene loss observed in *Zygophyllum* spp. may be related to the evolutionary history of the subfamily (Zygophylloideae); this point is interesting, and we recommend future study of different species in the family Zygophyllaceae. We also recommend that future studies include species from subfamilies Morkillioideae, Seetzenioideae and Larreoideae in Zygophyllaceae.

The Ka/Ks value is usually used for evaluating sequence variations in different species or taxonomical species with unknown evolutionary status and to detect substitution, selection and beneficial mutations in genes under selective pressure [75]. The values of synonymous (Ks) and nonsynonymous (Ka) substitutions and the Ka/Ks ratio showed that 14 protein-coding genes (*atpF, ndhG, petB, petD, psaI, psbH, psbT, rpl2, rps14, rps4, rps7, ycf4, rpl23,* and *matK*) were under positive selection and may have a faster evolutionary rate [57]. Most of these genes play a role in maintaining the efficiency of photosynthesis. In the current study, the *ycf4* gene was under positive selection. *ycf4* is located in the thylakoid membrane and involved in the assembly of the photosystem I complex [76], it is possible that *ycf4* has high substitution rates in arid plant species. Further research is required to investigate the use of these regions in detecting phylogenetic relationships among Zygophyllaceae species.

The plastome consists of many highly efficient genes that may resolve phylogenetic questions at different levels of angiosperm taxonomy [77, 78]. In this study, we found that *B. aegyptiaca* was distantly related to the family Simaroubaceae. This provides additional evidence to confirm the current position of *B. aegyptiaca* in the family Zygophyllaceae, as suggested by previous studies [21, 22, 25]. The chloroplast phylogenetic tree showed a strong relationship among Zygophyllaceae species. *B. aegyptiaca* and *T. terrestris* formed a group representing the Tribuloideae subfamily, *L. tridentata* and *G. angustifolium* (within the same branch) represent the Larreoideae subfamily, while *Z. mongolica, Z. fabago*, and *Z. xanthoxylon* were part of the same group and represent the Zygophylloideae subfamily. These findings broadly support the previous results of [1, 21].

## Materials and methods

### Sample collection and DNA extraction

Fresh leaves of *B. aegyptiaca* were collected from the Wadi Fatima (Al-Jamoum) Makkah district (21° 38′ 49.6“ N, 39° 41’ 49.3” E) in Saudi Arabia. The plants were identified by Dr. Widad S. Al-Juhani, assistant professor of taxonomy and supervisor of the herbarium in the Biology Department of Umm AlQura University, based on herbarium specimens and morphologies in relevant literature. A sample specimen was prepared and deposited in the herbarium of Umm Al-Qura University, Makkah, with accession number UQU072021. Samples of fresh leaves were dried in silica gel for DNA extraction. DNA was extracted from the silica gel-dried leaves of *B. aegyptiaca* using the CTAB Plant DNA extraction protocol [79].

### Library construction and De novo Genome sequencing

Library construction and sequencing using Illumina sequencing and read length 151 bp paired-ends were carried out by Macrogen (https://dna.macrogen.com/, Seoul, South Korea), with a final yield raw data of 3.5 Gb.

### Genome assembly and annotation

The FastQC tool was used to check raw read quality and remove adaptors. A Phred score above 30 was used. Clean reads were processed for genome assembly using NOVOPlasty 4.3.0 version [80] with kmer (K-mer = 33). The contig N50 value was high, and the plastome was assembled using the whole genomic sequence of *B. aegyptiaca*. *Tribulus terrestris* (NC_046758.1) was used as a reference in the assembly. Single contigs containing the plastome were generated. Gene prediction and annotation of the *B. aegyptiaca* chloroplast genome were carried out using the GeSeq tool [81], with default parameters and percent identity cut-off for protein-coding genes and RNAs set at ≥60 and ≤ 85, respectively. tRNA genes were identified with trnAscan-SE version 2.0 [82]. The annotated (gb) format sequence files were used to draw the circular chloroplast genome maps with the OGDRAW tool (Organellar Genome DRAW), version 1.3.1 [83]. The sequence of the chloroplast genome of *B. aegyptiaca* was deposited in the GenBank database with accession number (OL703321).

### Sequence Analysis

The relative synonymous codon usage (RSCU) values, base composition, and codon usage were analysed using MEGA software [84] version 11.0. Potential RNA editing sites present in the protein-coding genes were predicted by the PREP suite [85] with a cut-off value of 0.8.

### Repeat Analysis in the chloroplast Genome

The online software MIcroSAtellite (MISA) v2.1 [86] was used to identify simple sequence repeats (SSRs) in the chloroplast genome of *B. aegyptiaca* and five other species from the Zygophyllaceae family, namely, *Guaiacum angustifolium*, *Larrea tridentata, Tetraena mongolica, Tribulus terrestris,* and *Zygophyllum xanthoxylon*. Parameters eight, five, four and three repeats units were used: eight repeats for mononucleotide, five for dinucleotides, four for trinucleotides, three for each tetranucleotides, pentanucleotides, and hexanucleotides respectively.

In addition, REPuter [85] software was used with default settings to detect the size and location of long palindromic, forward, reverse, and complementary repeats in the *B. aegyptiaca* cp genome and the genomes of five species from Zygopyllaceae.

### Sequence divergence and boundary

Comparison of the genome of *B. aegyptiaca* with five chloroplast genome sequences from Zygophyllaceae (*G. angustifolium, L. tridentata, T. mongolica, T. terrestris,* and *Z. xanthoxylon*; GenBank accession numbers are shown in Table 4) was performed using the mVISTA program [87]; the annotation of *B. aegyptiaca* was used as a reference in the Shuffle-LAGAN mode. Furthermore, comparisons between the borders of the IR, SSC, and LSC regions were generated using IRSCOPE [88].

**Table 4:** Accession numbers of chloroplast genome analysed in the study

Orders	Family	Accession Number	Organism
Zygophyllales	Zygophyllaceae	OL703321	*Balanites aegyptiaca* (L.) Delile
		MK265246.1	*Tetraena mongolica* Maxim. *synonym of Zygophyllum mongolicum* (Maxim.) Christenh. & Byng
		MT796492.1	*Zygophyllum xanthoxylon* (Bunge) Maxim.
		NC_028023.1	*Larrea tridentata* (DC.) Coville
		NC_043796.1	*Guaiacum angustifolium* Engelm*.*
		NC_046758.1	*Tribulus terrestris* L.
	Krameriaceae	NC_043800.1	*Krameria bicolor* S.Watson
		NC_043801.1	*Krameria lanceolata* Torr.
Santalales	Olacaceae	MK764537.1	*Malania oleifera* Chun & S.K.Lee
	Schoepfiaceae	NC_034228.1	*Schoepfia jasminodora* Siebold & Zucc.
Sapindales	Anacardiaceae	NC_035973.1	*Spondias mombin* L.
	Burseraceae	NC_036978.1	*Commiphora wightii* (Arn.) Bhandar
	Meliaceae	NC_023792.1	*Azadirachta indica* A.Juss.
	Nitrariaceae	MH457633.1	*Nitraria tangutorum* Bobrov
		MW307830.1	*Peganum harmala* L.
	Rutaceae	NC_024929.1	*Citrus aurantiifolia* (Christm.) Swingle
	Sapindaceae	NC_040106.1	*Acer sino-oblongum* F.P.Metcalf
	Simaroubaceae	NC_030482.1	*Leitneria floridana* Chapm.
		NC_037696.1	*Ailanthus altissima* (Mill.) Swingle
Outgroup	Malvaceae	MK860036.1	*Malva parviflora* L.
		NC_049129.1	*Malva wigandii* (Alef.) M.F.Ray

### Characterisation of the substitution rate

The methods for estimating nonsynonymous and synonymous substitution rates (Ka and Ks), selection, and beneficial mutations among protein-coding sequences followed. Nonsynonymous (Ka) substitution, synonymous (Ks) substitution, and Ka/Ks ratios were calculated to detect variable mutation rates across chloroplast genom sequence, that contain an important information related to evolutionary history in *B. aegyptiaca* compared with those in the five aforementioned Zygophyllaceae species. We employed Ka/Ks Calculator version 2.0 [75] with default parameters, and the Nei and Gojobori substitution model was used.

### Phylogenetic Analysis

Phylogenetic analysis was conducted based on the cp genome sequences of the members of the Zygophyllales order, including cp genome sequences available in GenBank of species from the families Zygophyllaceae and Krameriaceae.

Because an older taxonomy placed *Balanites aegyptiaca* in the orders Sapindales and Santalales, the current phylogenetic analysis included sequesters of the cp genome of some species belonging to these orders. Information on species names, families, and GenBank accessions is available in Table 4. Two species from the Malvaceae family (*Malva parviflora* and *Malva wigandii*) were used as an outgroup.

All the common genes from the organisms were retrieved and the coding sequence CDS were joined in order. The sequences were further aligned using MAFFT version 7.475 [89]. Then phylogenetic trees were reconstructed based on the maximum parsimony (MP) method using 1000 bootstrap values and Mega software [84] version 11.0. The MP search method consisted of subtree-pruning-regrafting, with the number of initial trees (random addition) set at 10 and the number of threads set at 5.

The optimal evolutionary model was the GTR + I + G model, as calculated by ModelFinder using [90] Akaike’s information criterion (AIC) [91]. Bayesian inference analysis (BI) was conducted using MrBayes v. 3.2.6 [92, 93] in CIPRES Science Gateway 3.3 [94]. BI analysis included two separate runs; each of four Markov chain Monte Carlo chains was run for 10 million generations with sampling every 10,000 generations. Trees from the first 25% of the sampled generations were discarded as burn-in. The convergence of the runs was tested by using the effective sample size (ESS), calculated with Tracer v1.7.1 [95], with ESS values greater than 200 for all parameters considered good evidence. The majority rule (> 50%) consensus tree of BI was visualised using FigTree 1.4.3 [96].

## Conclusions

The aim of the present research was to provide the complete chloroplast genome of *B. aegyptiaca*, a plant in subfamily Tribuloideae and family Zygophyllaceae, which has medical and nutritional importance and plays a key role in ecosystem conservation in arid lands. We compared the cp genomes of available genera and species in the Zygopyllaceae family to assess the systematic relationships within the family and between related families as well as the genome conservation state.

This study confirmed the taxonomic status of the species *B. aegyptiaca* as a member of the Zygophyllaceae family. This study did not record loss in the chloroplast genome of *B. aegyptiaca,* as mentioned for species in subfamily Zygophylloideae. However, plastomes vary mainly at the SC/IR boundary among members of Zygophyllaceae, and there is clear genome reduction and gene loss in some species of Zygopyllaceae. Thus, we recommended further study to investigate changes that could have occurred in the structure of the genome during the evolutionary history of the family. It is necessary that future studies also include samples from subfamilies Morkillioideae, Seetzenioideae and Larreoideae in Zygophyllaceae.

## Supplementary Information

**Additional file 1.**

## Data Availability

The data presented in this study are available in this article and Supplementary Material. The complete chloroplast genome sequence of *Balanites aegyptiaca* was deposited in GenBank at https://www.ncbi.nlm.nih.gov, (accession numbers: OL703321).

## Abbreviations

Cp: Chloroplast
LSC: Large single copy region
SSC: Small single copy region
IR: Inverted repeat
RSCU: Relative synonymous codon usage
SSR: Simple sequence repeats
IGS: Intergenic spacer
CNS: Conserved non coding sequence
cpSSRs: Chloroplast simple sequence repeats
RAPD: Random Amplified Polymorphic DNA
MP: Maximum Parsimony
BP: Bootstrap percentage
BI: Bayesian Inference
PP: Posterior probability
Ks: Synonymous
Ka: Non-synonymous
CTAB: Cetrimonium bromide
PCR: Polymerase chain reaction
