# Supplementary material for: Complete chloroplast genome of the desert date (Balanites aegyptiaca (L.) Del. comparative analysis, and phylogenetic relationships among the members of Zygophyllaceae
Source: BMC Genomics. 2022 Aug 31;23:626. doi: 10.1186/s12864-022-08850-9 (PMC9434970; doi:10.1186/s12864-022-08850-9)
Supplement: Supplementary file 1 — Additional file 1. [file 12864_2022_8850_MOESM1_ESM.docx]

**Table S1:** Genes present in the plastome of *Balanites aegyptiaca*.

| **Category** | **Group of genes** | **Name of genes** |
| --- | --- | --- |
| **RNA genes** | Ribosomal RNA genes (rRNA) | Rrn4.5^a^, rrn16^a^, rrn5^a^, rrn23^a^ |
|  | Transfer RNA genes (tRNA) | trnr-UCU, trnC-GCA, trnT-GGU, trnG-GCC, trnS-GGA, trnF-GAA, trnV-GAC^+^,^a^, trnR-ACG^a^, trnL-UAG, trnN-GUU^a^, trnL-CAA^a^, trnM-CAU^a^, trnH-GUG^a^, trnP-UGG, trnW-CCA, trnT-UGU, trnS-UGA, trnE-UUC, trnY-GUA, trnD-GUC,trnS-GCU,trnQ-UUG,trnA-UGC^+^,a,trnI-CAUa,trnI-GAU^+^,a,trnG-UCC^+^,trnK-UUU^+^,trnL-UAA^+^ |
| **Ribosomal proteins** | Small subunit of ribosome | rps11, rps12^++,a^, rps14, rps15, rps16^+^, rps18, rps19, rps2, rps3, rps4, rps7^a^, rps8 |
| **Transcription** | Large subunit of ribosome | rpl14, rpl2^+,a^, rpl20, rpl22, rpl23^a^, rpl33, rpl36 |
|  | DNA dependent RNA polymerase | rpoA, rpoB, rpoC1^+^, rpoC2 |
| **Protein genes** | Photosystem I | psaA, psaB, psaC, psaI, psaJ |
|  | Photosystem II | psbA, psbB, psbC ,psbD, psbE, psbF, psbH, psbI, psbJ, psbK, psbM, psbT, psbZ |
|  | Subunit of cytochrome | petA, petB+, petD+, petG, petL, petN |
|  | Subunit of synthase | atpA, atpB, atpE, atpF^+^, atpH, atpI |
|  | Large subunit of RUBISCO | rbcL |
|  | NADH Dehydrogenase | ndhA^+^, ndhB^+,a^, ndhC, ndhD, ndhE, ndhF, ndhG, ndhH, ndhI, ndhJ, ndhK |
|  | Chloroplast envelope membrane protein | cemA |
| **Other genes** | Maturase | matK |
|  | Subunit acetyl-coA carboxylase | accD |
|  | C-type cytochrome synthesis | ccsA |
|  | protease | clpP^++^ |
|  | Component of TIC complex | ycf1 |
|  | Unkown Conserved open reading frames | ycf4, ycf2^a^ |

^+^ Gene with one intron, ^++^ Gene with two intron and ^a^ Gene with copies

**Table S2:** Codon-anticodon recognition patterns and codon usage in the *Balanites aegyptiaca* chloroplast genome.

| **Codon** | **Amino acid** | **RSCU** | **tRNA** | **Codon** | **Amino Acid** | **RSCU** | **tRNA** |
| --- | --- | --- | --- | --- | --- | --- | --- |
| UUU | Phe | 1.384 | trnF-GAA | UAU | Tyr | 1.656 | trnY-GUA |
| UUC | Phe | 0.616 |  | UAC | Tyr | 0.344 |  |
| UUA | Leu | 1.262 | trnL-UAA | UAA | Stop | 1.56 |  |
| UUG | Leu | 0.738 | trnL-CAA | UAG | Stop | 0.76 |  |
| CUU | Leu | 1.815 | trnL-UAG | CAU | His | 1.551 | trnH-GUG |
| CUC | Leu | 0.475 |  | CAC | His | 0.449 |  |
| CUA | Leu | 1.167 |  | CAA | Gln | 1.577 | trnQ-UUG |
| CUG | Leu | 0.543 |  | CAG | Gln | 0.423 |  |
| AUU | Ile | 1.535 | trnI-GAU | AAU | Asn | 1.59 | trnN-GUU |
| AUC | Ile | 0.516 |  | AAC | Asn | 0.41 |  |
| AUA | Ile | 0.949 | trnI-CAU | AAA | Lys | 1.555 | trnK-UUU |
| AUG | Met | 1 | trnM-CAU | AAG | Lys | 0.445 |  |
| GUU | Val | 1.458 | trnV-GAC | GAU | Asp | 1.619 | trnD-GUC |
| GUC | Val | 0.463 |  | GAC | Asp | 0.381 |  |
| GUA | Val | 1.557 |  | GAA | Glu | 1.557 | trnE-UUC |
| GUG | Val | 0.522 |  | GAG | Glu | 0.443 |  |
| UCU | Ser | 1.623 | trnS-GGA | UGU | Cys | 1.595 | trnC-GCA |
| UCC | Ser | 0.823 |  | UGC | Cys | 0.405 |  |
| UCA | Ser | 1.128 |  | UGA | Stop | 0.68 |  |
| UCG | Ser | 0.426 | trnS-UGA | UGG | Trp | 1 | trnW-CCA |
| CCU | Pro | 1.673 | trnP-UGG | CGU | Arg | 1.642 | trnR-ACG |
| CCC | Pro | 0.754 |  | CGC | Arg | 0.401 | trnR-UCU |
| CCA | Pro | 1.115 |  | CGA | Arg | 1.545 |  |
| CCG | Pro | 0.458 |  | CGG | Arg | 0.412 |  |
| ACU | Thr | 1.702 |  | AGA | Arg | 1.483 |  |
| ACC | Thr | 0.642 |  | AGG | Arg | 0.517 |  |
| ACA | Thr | 1.319 | trnT-GGU | AGU | Ser | 1.575 | trnS-GCU |
| ACG | Thr | 0.337 | trnT-UGU | AGC | Ser | 0.425 |  |
| GCU | Ala | 1.875 | trnA-UGC | GGU | Gly | 1.361 |  |
| GCC | Ala | 0.58 |  | GGC | Gly | 0.352 | trnG-GCC |
| GCA | Ala | 1.127 |  | GGA | Gly | 1.664 |  |
| GCG | Ala | 0.418 |  | GGG | Gly | 0.623 | trnG-UCC |

**Table S3:** The predicted RNA editing sites in *Balanites aegyptiaca* chloroplast genome.

| **Gene** | **Nucleotide Position** | **Aminoacid Position** | **Codon** | **Amino acid** | **Score** |
| --- | --- | --- | --- | --- | --- |
| accD | 539 | 180 | TCG (S) => TTG (L) | S => L | 0.8 |
|  | 1024 | 342 | CAC (H) => TAC (Y) | H => Y | 1 |
| atpA | 914 | 305 | TCA (S) => TTA (L) | S => L | 1 |
|  | 1148 | 383 | TCA (S) => TTA (L) | S => L | 1 |
| atpF | 92 | 31 | CCA (P) => CTA (L) | P => L | 0.86 |
| clpP | 559 | 187 | CAT (H) => TAT (Y) | H => Y | 1 |
| matK | 532 | 178 | CTT (L) => TTT (F) | L => F | 0.86 |
|  | 1184 | 395 | TCA (S) => TTA (L) | S => L | 0.86 |
| ndhA | 341 | 114 | TCA (S) => TTA (L) | S => L | 1 |
|  | 566 | 189 | TCA (S) => TTA (L) | S => L | 1 |
| ndhB | 149 | 50 | TCA (S) => TTA (L) | S => L | 1 |
|  | 467 | 156 | CCA (P) => CTA (L) | P => L | 1 |
|  | 542 | 181 | ACG (T) => ATG (M) | T => M | 1 |
|  | 586 | 196 | CAT (H) => TAT (Y) | H => Y | 1 |
|  | 611 | 204 | TCA (S) => TTA (L) | S => L | 0.8 |
|  | 737 | 246 | CCA (P) => CTA (L) | P => L | 1 |
|  | 746 | 249 | TCT (S) => TTT (F) | S => F | 1 |
|  | 830 | 277 | TCA (S) => TTA (L) | S => L | 1 |
|  | 836 | 279 | TCA (S) => TTA (L) | S => L | 1 |
|  | 1112 | 371 | TCA (S) => TTA (L) | S => L | 1 |
|  | 1255 | 419 | CAT (H) => TAT (Y) | H => Y | 1 |
|  | 1481 | 494 | CCA (P) => CTA (L) | P => L | 1 |
| ndhD | 29 | 10 | ACG (T) => ATG (M) | T => M | 1 |
|  | 74 | 25 | TCC (S) => TTC (F) | S => F | 0.8 |
|  | 410 | 137 | TCA (S) => TTA (L) | S => L | 1 |
|  | 626 | 209 | TCA (S) => TTA (L) | S => L | 1 |
|  | 1325 | 442 | TCA (S) => TTA (L) | S => L | 0.8 |
|  | 1432 | 478 | CTT (L) => TTT (F) | L => F | 0.8 |
| ndhF | 290 | 97 | TCA (S) => TTA (L) | S => L | 1 |
|  | 1172 | 391 | GCG (A) => GTG (V) | A => V | 0.8 |
|  | 2002 | 668 | CTT (L) => TTT (F) | L => F | 1 |
| ndhG | 314 | 105 | ACA (T) => ATA (I) | T => I | 0.8 |
| rpoB | 338 | 113 | TCT (S) => TTT (F) | S => F | 1 |
|  | 551 | 184 | TCA (S) => TTA (L) | S => L | 1 |
|  | 566 | 189 | TCG (S) => TTG (L) | S => L | 1 |
| rpoC1 | 41 | 14 | TCA (S) => TTA (L) | S => L | 1 |
| rpoC2 | 3749 | 1250 | TCA (S) => TTA (L) | S => L | 0.86 |
| rps14 | 80 | 27 | TCA (S) => TTA (L) | S => L | 1 |
| rps16 | 212 | 71 | TCA (S) => TTA (L) | S => L | 0.83 |
| rps2 | 248 | 83 | TCA (S) => TTA (L) | S => L | 1 |
| rps8 | 182 | 61 | TCA (S) => TTA (L) | S => L | 0.86 |

**Table S4:** Repeat sequences present in the chloroplast genome of *Balanites aegyptiaca*.

| **SN** | **Repeat Size** | **Repeat Position 1** | **Repeat Type** | **Repeat Location 1** | **Repeat Position 2** | **Repeat Location 2** | **E-value** |
| --- | --- | --- | --- | --- | --- | --- | --- |
| 1 | 42 | 101208 | F | IGS | 122106 | ndhA | 3.53E-16 |
| 2 | 42 | 122106 | P | ndhA | 141112 | IGS | 3.53E-16 |
| 3 | 38 | 61036 | P | IGS | 61036 | accD | 9.04E-14 |
| 4 | 31 | 82896 | F | IGS | 82927 | IGS | 1.48E-09 |
| 5 | 30 | 8419 | P | trnS-GCU | 47241 | trnS-GGA | 5.92E-09 |
| 6 | 30 | 38047 | R | IGS | 38047 | IGS | 5.92E-09 |
| 7 | 30 | 129498 | P | IGS | 129498 | IGS | 5.92E-09 |
| 9 | 28 | 73567 | P | clpP | 73605 | clpP | 9.47E-08 |
| 8 | 28 | 30466 | P | IGS | 30529 | IGS | 9.47E-08 |
| 12 | 25 | 122534 | P | ndhA | 122561 | ndhA | 6.06E-06 |
| 10 | 25 | 268 | P | IGS | 317 | IGS | 6.06E-06 |
| 11 | 25 | 38174 | F | IGS | 38198 | IGS | 6.06E-06 |
| 13 | 24 | 10685 | P | IGS | 10712 | IGS | 2.43E-05 |
| 14 | 24 | 38206 | F | IGS | 38225 | IGS | 2.43E-05 |
| 15 | 24 | 38231 | P | IGS | 38231 | IGS | 2.43E-05 |
| 16 | 24 | 53473 | F | IGS | 53497 | IGS | 2.43E-05 |
| 17 | 24 | 70105 | P | IGS | 70148 | IGS | 2.43E-05 |
| 18 | 23 | 10410 | P | IGS | 10440 | IGS | 9.70E-05 |
| 19 | 23 | 10844 | R | IGS | 10844 | IGS | 9.70E-05 |
| 20 | 23 | 38926 | P | IGS | 38961 | IGS | 9.70E-05 |
| 21 | 22 | 70716 | F | IGS | 84100 | IGS | 3.88E-04 |
| 22 | 21 | 8425 | F | trnS-GCU | 37128 | trnS-UGA | 1.55E-03 |
| 26 | 21 | 37128 | P | trnS-UGA | 47244 | trnS-GGA | 1.55E-03 |
| 28 | 21 | 93892 | F | ycf2 | 93916 | ycf2 | 1.55E-03 |
| 29 | 21 | 93892 | P | ycf2 | 148425 | ycf2 | 1.55E-03 |
| 30 | 21 | 93916 | P | ycf2 | 148449 | ycf2 | 1.55E-03 |
| 33 | 21 | 148425 | F | ycf2 | 148449 | ycf2 | 1.55E-03 |
| 23 | 21 | 8665 | R | IGS | 8665 | IGS | 1.55E-03 |
| 24 | 21 | 8669 | R | IGS | 48368 | IGS | 1.55E-03 |
| 25 | 21 | 28323 | P | IGS | 81825 | IGS | 1.55E-03 |
| 27 | 21 | 65473 | P | IGS | 83380 | IGS | 1.55E-03 |
| 31 | 21 | 118909 | P | IGS | 118947 | IGS | 1.55E-03 |
| 32 | 21 | 119002 | R | IGS | 119002 | IGS | 1.55E-03 |
| 39 | 20 | 37196 | P | trnS-UGA | 47183 | trnS-GGA | 6.21E-03 |
| 41 | 20 | 52945 | R | ndhC | 52945 | ndhC | 6.21E-03 |
| 34 | 20 | 4361 | R | IGS | 4361 | IGS | 6.21E-03 |
| 35 | 20 | 4580 | R | IGS | 4580 | IGS | 6.21E-03 |
| 36 | 20 | 7339 | R | IGS | 70713 | IGS | 6.21E-03 |
| 37 | 20 | 7392 | R | IGS | 7392 | IGS | 6.21E-03 |
| 38 | 20 | 8913 | R | IGS | 58951 | IGS | 6.21E-03 |
| 40 | 20 | 49124 | R | IGS | 49124 | IGS | 6.21E-03 |
| 42 | 20 | 119008 | R | IGS | 119008 | IGS | 6.21E-03 |
| 43 | 19 | 226 | P | IGS | 246 | IGS | 2.48E-02 |
| 44 | 19 | 4557 | C | IGS | 68997 | IGS | 2.48E-02 |
| 45 | 19 | 4576 | F | IGS | 83377 | IGS | 2.48E-02 |
| 46 | 19 | 4580 | R | IGS | 4580 | IGS | 2.48E-02 |
| 47 | 19 | 4580 | F | IGS | 4581 | IGS | 2.48E-02 |
| 48 | 19 | 4581 | R | IGS | 4581 | IGS | 2.48E-02 |
| 49 | 19 | 4770 | F | IGS | 37392 | IGS | 2.48E-02 |

| **Species** | **Description** |
| --- | --- |
| *Tribulus terrestris* | Stem cylindrical green to red, covered with a strong pubescence of white hair. The leaves are compound and opposite. Flowers are solitary; corolla has five yellow petals obovate and numerous stamens. Fruits consist of 5 triangular segments arranged in a star and become hard at maturity; each segment contains 3-5 seeds [97]. |
| *Tetraena mongolica.*  *synonym of*  *Zygophyllum mongolicum* | Shrubs or herbs; leaves are opposite. The flower has five petals and five sepals, rarely four, usually tube-like with white to pale orange petals, ripe fruits are variable in shape [98]. |
| *Zygophyllum xanthoxylon*  *synonym of*  *Zygophyllum xanthoxylum* | Shrubs with zigzag branches and spiny-pointed, bright grey bark. Leaves are linear-oblong or linear. Four flowers sepals and 4 light yellow petals. Fruit is a spherical capsule [99]. |
| *Guaiacum angustifolium* | Branched shrub or small tree. Leaves pinnately compound; flowers small blue to purple. Fruit capsule with one or two lobes, containing a single shiny bean-like seed [100, 101]. |
| *Larrea tridentata* | Evergreen shrub, the leaves of creosote bush are thick, resinous, and strongly scented. Flowers are solitary with five carpels densely covered by long trichomes. Fruits are globose [102-104]. |

**Table S5:** Description of species mentioned in this study.

1. Le B, Merlier T, Adventrop H. Les adventices d'Afrique soudano-sahélienne. Cirad, Montpellier, France.1995.
2. Beier BA, Chase MW, Thulin M. Phylogenetic relationships and taxonomy of subfamily Zygophylloideae (Zygophyllaceae) based on molecular and morphological data. Plant Syst. Evol. 2003, 240 (1–4): 11–39, doi:10.1007/s00606-003-0007-0, S2CID 9299742.
3. Flora of China, family list, Zygophyllaceae, Zygophyllum xanthoxylo. FOC. 2022, 11: 45-46. <http://www.efloras.org>.
4. Richardson A. Plants of the Rio Grande Delta. University of Texas Press. 1995: 123.
5. Powell AM. Trees and Shrubs of the Trans-Pecos and Adjacent Areas. University of Texas Press.1998 :203–204.
6. Bainbridge DA. Virginia, Ross A. Restoration in the Sonoran Desert of California. Restoration and Management Notes.1990, 8(1): 3-14.
7. Kearney TH, Peebles RH, Howell JT, McClintock E. Arizona flora. 2d ed. Berkeley, CA: University of California Press. 1960:1085.
8. Maddox JC, Carlquist S. Wind dispersal in Californian desert plants: experimental studies and conceptual considerations. Aliso. 1985, 11(1): 77-96.

**Table S6:** length of four-region LSC, SSC and IR and complete chloroplast genomes used in comparison.

| **Species** | **Accession** | **Size(bp)** | **LSC (bp)** | **SSC (bp)** | **IR (bp)** |
| --- | --- | --- | --- | --- | --- |
| *Balanites aegyptiaca* | OL703321 | 155800 | 86562 | 18102 | 25568 |
| *Tetraena mongolica synonym of Zygophyllum mongolicum* | MK265246.1 | 106288 | 80458 | 17200 | 4315 |
| *Zygophyllum xanthoxylon* | MT796492.1 | 109577 | 83735 | 15674 | 5084 |
| *Larrea tridentata* | NC_028023.1 | 136194 | 81536 | 15958 | 19350 |
| *Guaiacum angustifolium* | NC_043796.1 | 130809 | 81554 | 13767 | 17744 |
| *Tribulus terrestris* | NC_046758.1 | 158184 | 88878 | 17622 | 25842 |


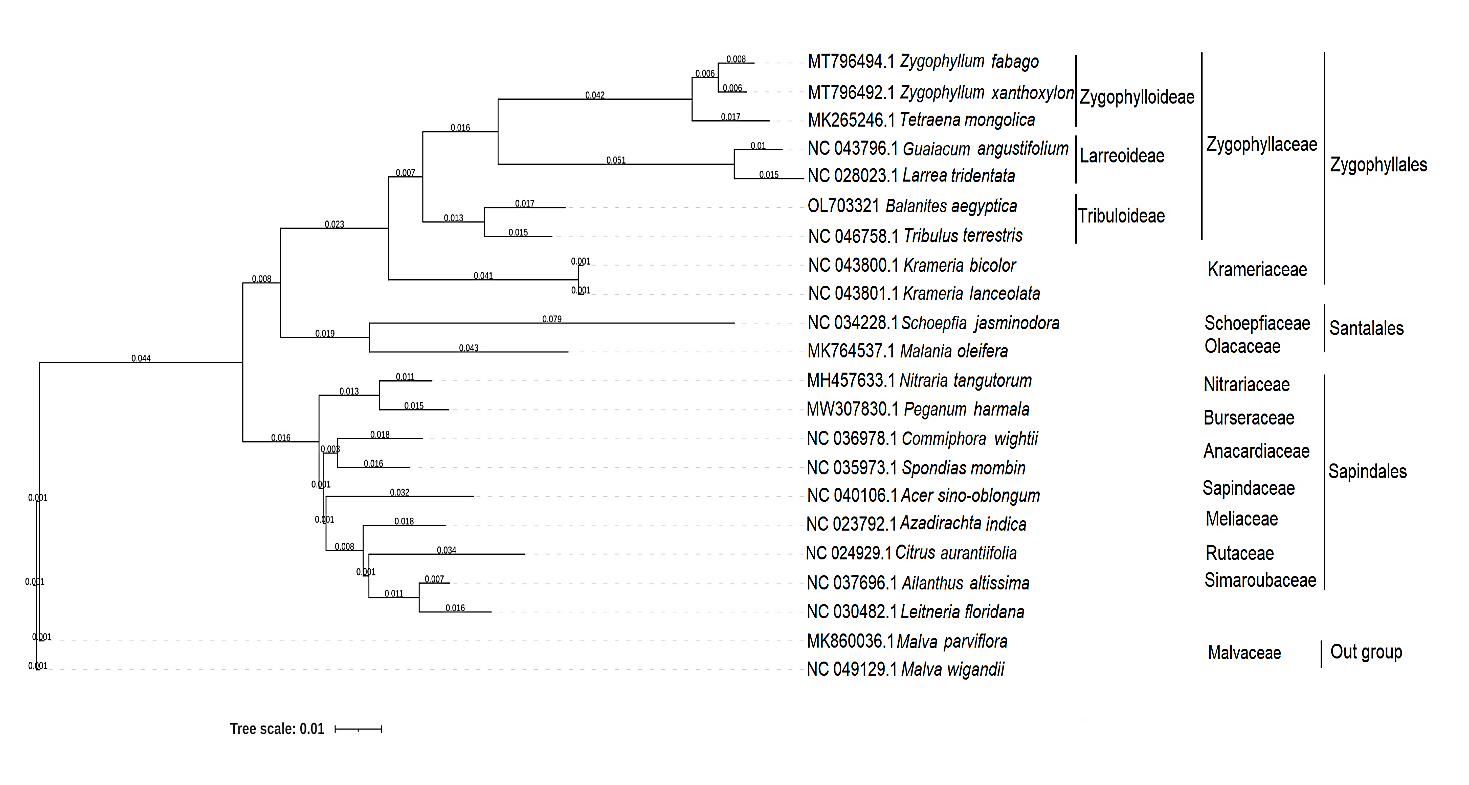


**Figure A1:** Phylogenetic tree (with branch lengths) construction inferred from the coding sequence (CDS) of 20 taxa, using Bayesian Inference (BI) and Maximum Parsimony (MP) methods. The tree shows the relationships between Zygophyllales (Krameriaceae & Zygophyllaceae), Sapindales, and Santalales.
